# Serum KL-6 and eGFR predict everolimus-associated KL-6 elevation in heart transplant recipients: a retrospective observational study

**DOI:** 10.1186/s40780-026-00603-0

**Published:** 2026-07-01

**Authors:** Yuhei Suzuki, Toyohito Oriyama, Takehito Yamamoto, Masaru Hatano, Eisuke Amiya, Masaki Tsuji, Chie Bujo, Norihiko Takeda, Masahiko Ando, Minoru Ono, Tappei Takada

**Affiliations:** 1 Department of Pharmacy, The University of Tokyo Hospital, 7-3-1 Hongo, Bunkyo-ku, Tokyo, 113-8655 Japan; 2 Department of Cardiovascular Medicine, Graduate School of Medicine, The University of Tokyo, 7-3-1 Hongo, Tokyo, 113-8655 Japan; 3 Department of Cardiovascular Surgery, Graduate School of Medicine, The University of Tokyo, 7-3-1 Hongo, Bunkyo-ku, Tokyo, 113-8655 Japan

**Keywords:** Everolimus, Immunosuppressive agent, Heart transplant, KL-6, eGFR, Drug-associated, Lung injury

## Abstract

**Background:**

Everolimus (EVR), which is widely used in heart transplant recipients, has been associated with drug-induced lung injury. Although EVR-associated lung injury has been reported in patients with malignancies and recipients of other solid organ transplants, its incidence and risk factors in heart transplant recipients remain unclear. Krebs von den Lungen-6 (KL-6) is a serum biomarker widely used in the assessment of interstitial lung diseases and may reflect pulmonary involvement, including drug-induced lung injury. This study aimed to determine the incidence and risk factors for KL-6 elevation after EVR administration in heart transplant recipients.

**Methods:**

This retrospective observational study included patients who received a heart transplant at the University of Tokyo Hospital from June 2006 to April 2021. The patients were categorized into two groups: those who received EVR after heart transplantation (EVR group) and those who did not (non-EVR group). Multivariable logistic regression analysis was performed in the EVR group to identify independent risk factors for KL-6 elevation (defined as a peak level ≥ 500 U/mL). Receiver operating characteristic (ROC) analysis was used to determine optimal cutoff values, and a composite risk score was constructed. Event-free survival was evaluated using the Kaplan–Meier method.

**Results:**

Seventy-three patients were included (58 in the EVR group and 15 in the non-EVR group). Peak serum KL-6 levels were significantly higher in the EVR group than in the non-EVR group (320 [235–509] vs. 157 [127–263] U/mL, *p* = 0.002). KL-6 elevation occurred in 27.6% and 6.7% of patients in the EVR and non-EVR groups, respectively. Within the EVR group, higher baseline KL-6 levels and lower eGFR were independently associated with KL-6 elevation. ROC analysis identified cutoff values of baseline KL-6 > 268 U/mL and eGFR < 60 mL/min/1.73 m^2^. Higher composite risk scores were associated with a greater incidence of KL-6 elevation, and patients with both risk factors experienced earlier KL-6 elevation.

**Conclusions:**

EVR administration was associated with KL-6 elevation in heart transplant recipients. Elevated baseline KL-6 levels and impaired renal function before EVR initiation were independent predictors of KL-6 elevation. Assessment of these parameters may help earlier detection of EVR-associated KL-6 elevation.

**Supplementary Information:**

The online version contains supplementary material available at https://doi.org/10.1186/s40780-026-00603-0.

## Background

Everolimus (EVR), an inhibitor of mammalian target of rapamycin (mTOR), is widely used as an immunosuppressive agent in patients undergoing solid organ transplantation (SOT). Previous reports have shown that EVR enables a dose reduction of calcineurin inhibitors (cyclosporin and tacrolimus), which are associated with renal dysfunction; thereby decreasing the risk of nephrotoxicity without increasing the risk of rejection [1–3]. In addition, EVR reportedly inhibits the development of cytomegalovirus infection and the progression of cardiac allograft vasculopathy [4–7]. Owing to these benefits, EVR is now widely used in patients who underwent heart transplantation.

Drug-induced lung injury (LI) is a well-recognized adverse event associated with EVR in cancer patients [8–11]. However, data on EVR-associated LI in SOT recipients are limited. Previous reports have documented that the frequency of EVR-associated LI ranges from 2.8% to 12.7% in kidney transplant recipients [12, 13]. A few case reports have described severe LI after EVR administration in heart transplant recipients but the incidence of EVR-associated LI in heart transplant recipients remains unclear [14–16].

In addition, elevated serum Krebs von den Lungen-6 (KL-6) levels and low estimated glomerular filtration rate (eGFR) before EVR administration have been identified as risk factors for EVR-associated LI in patients with cancer [17], however, the risk factors for EVR-associated LI in SOT recipients have not yet been identified. Considering the differences in EVR dosage and background characteristics between cancer patients and SOT recipients, it is clinically important to explore the risk factors for EVR-associated LI in SOT recipients to enable early detection and prevent its progression.

Although no reliable serum biomarker for the diagnosis of EVR-induced LI has been established, a previous study reported that elevated serum KL-6 is a possible surrogate biomarker for the diagnosis of EVR-induced LI [17]. Therefore, in this retrospective observational study, we aimed to determine the incidence of EVR-associated KL-6 elevation and identify its risk factors in heart transplant recipients.

## Methods

### Patients

This retrospective study included patients who received a heart transplant at the University of Tokyo Hospital from June 2006 to April 2021, were > 15 years of age at the time of transplantation, and had their serum KL-6 levels measured at least twice within March 2023. The exclusion criteria were as follows: 1) baseline or peak serum KL-6 levels were unavailable; 2) serum KL-6 levels were not measured within 2 years after EVR initiation or transplantation; and 3) the date of EVR initiation was unknown. The patients were categorized into two groups: those who received EVR after heart transplantation (EVR group) and those who did not (non-EVR group).

### Immunosuppression therapy

All transplant recipients received standard immunosuppressive therapy consisting of calcineurin inhibitors (tacrolimus or cyclosporine), mycophenolate mofetil, and prednisolone. During treatment, mycophenolate mofetil was switched to everolimus in some cases, whereas calcineurin inhibitors were continued in all cases. Prednisolone administration was gradually tapered and discontinued within one year. The main reasons for the introduction of everolimus included coronary allograft vasculopathy, acute cellular rejection, renal dysfunction, cytopenia, and gastrointestinal symptoms.

### Data collection and definition

The following data were collected from the electronic medical records of the patients: date of transplantation, calcineurin inhibitor medications, history of steroid pulse therapy and amiodarone therapy, smoking history, date of EVR initiation, clinical laboratory data at the date of heart transplantation and EVR initiation, baseline and peak serum KL-6 levels, and blood EVR concentration and dose at the time of peak serum KL-6 level. Hemodynamic parameters (mean right atrial pressure [mRAP] and mean pulmonary capillary wedge pressure [mPCWP]) were extracted from right heart catheterization (RHC) records obtained closest to the date of the peak KL-6 measurement after EVR initiation. Serum creatinine (Cre) was measured using an enzymatic method, and the eGFR was calculated using the formula developed by the Japanese Society of Nephrology:

eGFR (mL/min/1.73 m^2^) = 194 × serum creatinine^−1.094^ × age^−0.287^ × 0.739 (if female) [18].

In this study, baseline serum KL-6 levels were measured before EVR initiation in the EVR group and before or within six months of transplantation in the non-EVR group. Peak serum KL-6 level was defined as the highest KL-6 level observed after EVR initiation in the EVR group and at six months post-transplantation in the non-EVR group. Based on previous literature reporting a cutoff of 500 U/mL for diagnosing LI [19], we defined the event of “KL-6 elevation” as a peak serum KL-6 level ≥ 500 U/mL. Patients were classified into the KL-6 elevated group and KL-6 stable group according to whether their peak serum KL-6 levels were ≥ 500 U/mL or <500 U/mL, respectively.

### Statistical analysis

Data are expressed as median (interquartile range). Continuous and categorical variables were analyzed using a Mann-Whitney U test and a Fisher’s exact test, respectively. Multivariable logistic regression analysis was performed using factors with *p* values < 0.1 between the KL-6 elevated and KL-6 stable groups. Variables identified as independent risk factors using multivariable logistic regression analysis were subjected to a receiver operating characteristic (ROC) curve analysis to determine their cutoff values.

Risk scores for each patient were calculated using the cutoff values of the independent risk factors (baseline KL-6 level and eGFR). Each factor was assigned a score of 1 if its value exceeded the threshold, and a score of 0 otherwise. Patients were stratified into three groups according to their total risk scores. Event-free survival was estimated for the three risk score groups using the Kaplan-Meier method, and comparisons among the three groups were performed using the log-rank test with Bonferroni correction. Statistical analyses were performed using JMP® Pro16 (SAS Institute, Cary, NC, USA) and a *p* value of <0.05 was considered statistically significant.

## Results

### Patient characteristics

**Fig. 1:**
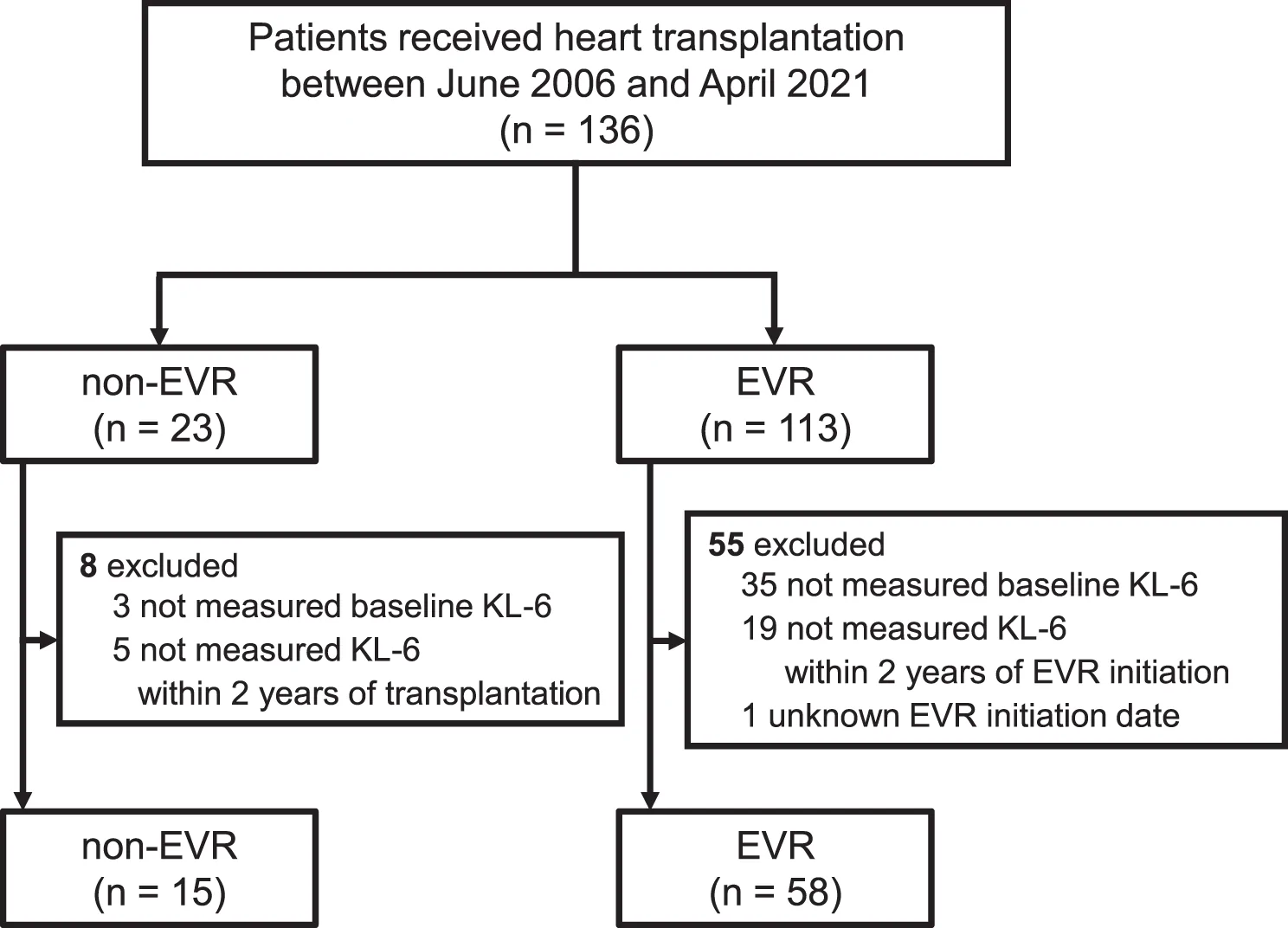
Flowchart of patient group classification EVR: everolimus, KL-6: Krebs von den Lungen-6

In this study, 136 patients were assessed for eligibility, and 73 patients (58 patients in the EVR group and 15 patients in the non-EVR group) were included in the final analysis (Fig. 1).

**Fig. 2:**
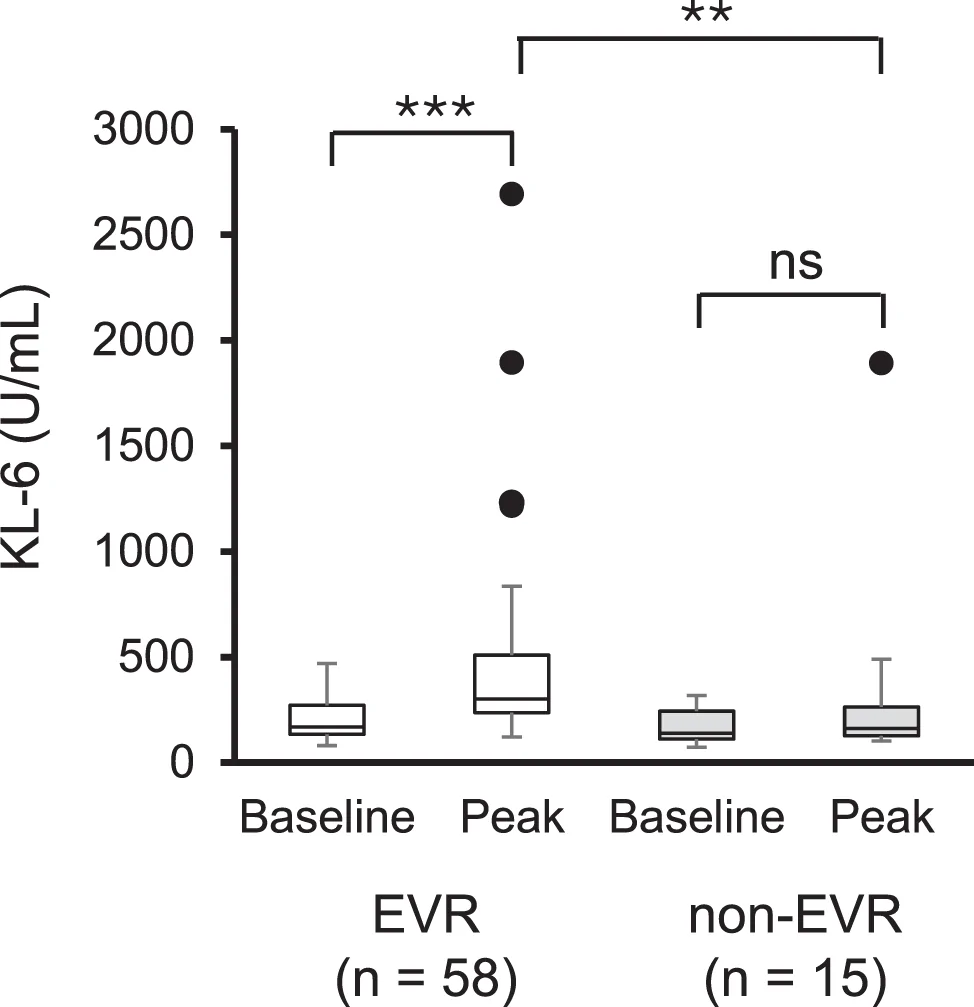
Changes in serum KL-6 levels in EVR and non-EVR groups. Box plots show baseline and peak serum KL-6 levels in patients receiving EVR (*n* = 58) and those not receiving EVR (non-EVR group, *n* = 15). In the EVR group, peak KL-6 levels were significantly higher than baseline levels, whereas no significant difference was observed between baseline and peak levels in the non-EVR group. Additionally, peak KL-6 levels were significantly higher in the EVR group compared to the non-EVR group. ns: not significant, **: *p* < 0.01, ***: *p* < 0.001. EVR: everolimus, KL-6: Krebs von den Lungen-6

Table 1 shows the patient demographics at the time of heart transplantation. The baseline serum KL-6 levels were similar between the two groups (169 [134–271] U/mL and 139 [111–244] U/mL in the EVR and non-EVR groups, respectively, *p* = 0.146). In contrast, peak serum KL-6 levels were significantly higher in the EVR group (320 [235–509] U/mL) than that in the non-EVR group (157 [127–263] U/mL) (*p* = 0.002) (Fig. 2). The proportion of patients who experienced a KL-6 elevation tended to be higher in the EVR group than that in the non-EVR group (27.6% [16/58] vs. 6.7% [1/15], *p* = 0.079). LDH levels were significantly lower in the EVR group (378 [274–476] U/L) than in the non-EVR group (496 [378–727] U/L) (*p* = 0.036). The median time to everolimus initiation was 116 [84–250] days after transplantation.

**Table 1:** Patient characteristics at the time of heart transplantation

	EVR (n = 58)	non-EVR (n = 15)	*p* **value**
Age [Years], median (IQR)	45 (32–50)	41 (28–47)	0.692
Male, n (%)	47 (81.0)	9 (60.0)	0.099
BMI [kg/m^2^], median (IQR)	21.1 (19.5–24.1)	20.9 (18.7–24.0)	0.919
History of smoking, n (%)	23 (39.7)	5 (33.3)	0.770
Calcineurin Inhibitors, n (%)			
Tacrolimus	32 (55.2)	10 (66.7)	
Ciclosporin	26 (44.8)	5 (33.3)	0.561
History of steroid pulse therapy, n (%)	31 (53.4)	5 (33.3)	0.247
History of amiodarone, n (%)	29 (50.0)	7 (46.7)	1.000
Laboratory data, median (IQR)			
AST [U/L]	29 (22–38)	31 (25–44)	0.452
ALT [U/L]	18 (11–25)	22 (12–32)	0.461
T-Bil [mg/dL]	0.9 (0.6–1.3)	0.8 (0.6–1.0)	0.320
LDH [U/L]	378 (274–476)	496 (378–727)	0.036
Albumin [g/dL]	4.4 (4.0–4.6)	4.3 (4.2–4.6)	0.800
CRP [mg/dL]	0.26 (0.11–0.86)	0.39 (0.20–1.09)	0.440
BUN [mg/dL]	15.1 (12.3–19.3)	15.6 (13.6–18.9)	0.859
Creatinine [mg/dL]	0.85 (0.73–1.03)	0.74 (0.66–1.01)	0.367
eGFR [mL/min/1.73 m^2^]	75.5 (62.9–92.7)	85.3 (55.8–99.0)	0.886
White blood cell [/µL]	7100 (6100–8150)	7500 (6200–8550)	0.657
Neutrophil [/µL]	3857 (2325–5060)	5872 (4485–7550)	0.100
Eosinophil [/µL]	39 (20–72)	64 (20–167)	0.723
Hemoglobin [g/dL]	12.8 (11.8–13.9)	12.3 (10.5–13.3)	0.100
Platelet [×10^4^/µL]	20.3 (16.7–25.9)	20.1 (17.8–22.9)	0.723
BNP [pg/mL]	141.3 (97.1–287.0)	149.9 (68.6–253.8)	0.790
Baseline KL–6 level [U/mL], median (IQR)^a^	169 (134–271)	139 (111–244)	0.146

EVR: everolimus, BMI: body mass index, AST: aspartate aminotransferase, ALT: alanine aminotransferase, T-bil; total bilirubin, LDH: lactate dehydrogenase, CRP: C-reactive protein, BUN: blood urea nitrogen, eGFR: estimated glomerular filtration rate, BNP: B-type natriuretic peptide, KL-6: Krebs von den Lungen-6, ^a^: Baseline KL-6 levels were defined as measured before the EVR initiation in the EVR group, and before or within 6 months after transplantation in the non-EVR group

The EVR group was divided into two subgroups for risk factor analysis: 16 patients were classified into the KL-6 elevated group and 42 patients into the KL-6 stable group, respectively. Table 2 shows the demographics of the patients at the time of EVR initiation. eGFR levels were significantly lower in the KL-6 elevated group compared to the KL-6 stable group (57.4 [43.9–65.0] vs. 64.3 [57.2–83.6] mL/min/1.73 m^2^, *p* = 0.035). Consistent with this, Cre levels were significantly higher in the KL-6 elevated group compared to the KL-6 stable group (1.20 [0.98–1.44] vs. 0.94 [0.81–1.13] mg/dL, *p* = 0.011). Baseline serum KL-6 levels were significantly higher in the KL-6 elevated group than in the KL-6 stable group (300 [152–371] vs. 167 [127–214] U/mL, *p* = 0.005). The proportion of female patients tended to be higher in the KL-6 elevated group than in the KL-6 stable group (37.5 vs. 11.9%, *p* = 0.055), and the age at heart transplantation tended to be lower in the KL-6 elevated group (33 [25 − 48] vs. 45 [36–51] years, *p* = 0.060). There were no significant differences in the history of amiodarone (43.8 vs. 52.4%, *p* = 0.770). As expected, peak serum KL-6 levels were significantly higher in the KL-6 elevated group than in the KL-6 stable group (597 [531–931] vs. 252 [199–334] U/mL, *p* < 0.001). RHC data obtained closest to the time of peak KL-6 measurement showed no significant differences in mRAP or mPCWP between the two groups, and no marked elevation suggestive of overt volume overload was observed (Table 3). Among 16 patients in KL-6 elevated group, 37.5% (6/16) of patients had a history of cytomegalovirus antigenemia, but no patients were diagnosed with cytomegalovirus pneumonitis at the time of peak KL-6.

**Table 2:** Patient characteristics at the time of EVR initiation in the EVR group

	KL-6 elevated (n = 16)	KL-6 stable (n = 42)	*p* **value**
Age [years], median (IQR)	33 (25–48)	45 (36–51)	0.060
Male, n (%)	10 (62.5)	37 (88.1)	0.055
BMI [kg/m^2^], median (IQR)	20.1 (16.8–23.5)	20.3 (18.6–22.2)	0.584
History of smoking, n (%)	3 (18.8)	20 (47.6)	0.071
Calcineurin Inhibitors, n (%)			
Tacrolimus	9 (56.3)	23 (54.8)	
Ciclosporin	7 (43.7)	19 (45.2)	1.000
History of steroid pulse therapy, n (%)	7 (43.8)	24 (57.1)	0.393
History of amiodarone, n (%)	7 (43.8)	22 (52.4)	0.770
Time to EVR initiation [days], median (IQR)	94 (80–181)	122 (88–335)	0.465
Laboratory data, median (IQR)			
AST [U/L]	22 (16–33)	19 (15–26)	0.308
ALT [U/L]	13 (10–34)	16 (12–25)	0.454
T-Bil [mg/dL]	0.5 (0.4–0.9)	0.6 (0.4–0.7)	0.937
LDH [U/L]	278 (235–300)	253 (199–328)	0.503
Albumin [g/dL]	4.0 (3.8–4.3)	4.3 (3.9–4.4)	0.145
CRP [mg/dL]	0.02 (0.02–0.10)	0.05 (0.02–0.18)	0.323
BUN [mg/dL]	18.7 (13.9–24.9)	18.7 (16.2–21.9)	0.855
Creatinine [mg/dL]	1.20 (0.98–1.44)	0.94 (0.81–1.13)	0.011
eGFR [mL/min/1.73 m^2^]	57.4 (43.9–65.0)	64.3 (57.2–83.6)	0.035
White blood cell [/µL]	5050 (2625–5725)	5050 (3700–6375)	0.645
Neutrophil [/µL]	4098 (2066–4594)	3729 (2414–5188)	0.781
Eosinophil [/µL]	31 (12–54)	41 (22–72)	0.356
Hemoglobin [g/dL]	11.8 (10.9–12.8)	11.9 (11.0–12.8)	0.525
Platelet [×10^4^/µL]	21.2 (19.7–26.9)	22.4 (18.9–27.1)	0.903
BNP [pg/mL]	102.8 (45.1–149.4)	91.5 (47.4–137.4)	0.862
Baseline KL–6 level [U/mL], median (IQR)^a^	300 (152–371)	167 (127–214)	0.005

EVR: everolimus, AST: aspartate aminotransferase, ALT: alanine aminotransferase, T-bil; total bilirubin, LDH: lactate dehydrogenase, CRP: C-reactive protein, BUN: blood urea nitrogen, eGFR: estimated glomerular filtration rate, BNP: B-type natriuretic peptide, KL-6: Krebs von den Lungen-6, ^a^: Baseline KL-6 levels were defined as those measured before the EVR initiation

**Table 3:** Clinical characteristics and outcomes at the time of peak KL-6 levels in the EVR group

	KL-6 elevated (n = 16)	KL-6 stable (n = 42)	*p* **value**
Peak KL–6 level [U/mL], median (IQR)	597 (531–931)	252 (199–334)	<0.001
EVR dose [mg], median (IQR)	0.9 (0.7–1.0)	1.0 (0.8–1.0)	0.586
EVR concentration [ng/mL], median (IQR)	6.5 (5.7–7.6)	5.6 (3.9–6.6)	0.115
Right heart catheterization data^a^			
mRAP [mmHg]	4 (2–5)	3 (2–5)	0.261
mPCWP[mmHg]	9 (7–10)	8 (6–10)	0.182

EVR: everolimus, KL-6: Krebs von den Lungen-6, mRAP: mean right atrial pressure, mPCWP: mean pulmonary capillary wedge pressure, ^a^: Hemodynamic parameters (mRAP and mPCWP) were obtained from right heart catheterization records at time points closest to the peak KL-6 measurement

## Risk factors for EVR-associated KL-6 elevation

**Fig. 3:**
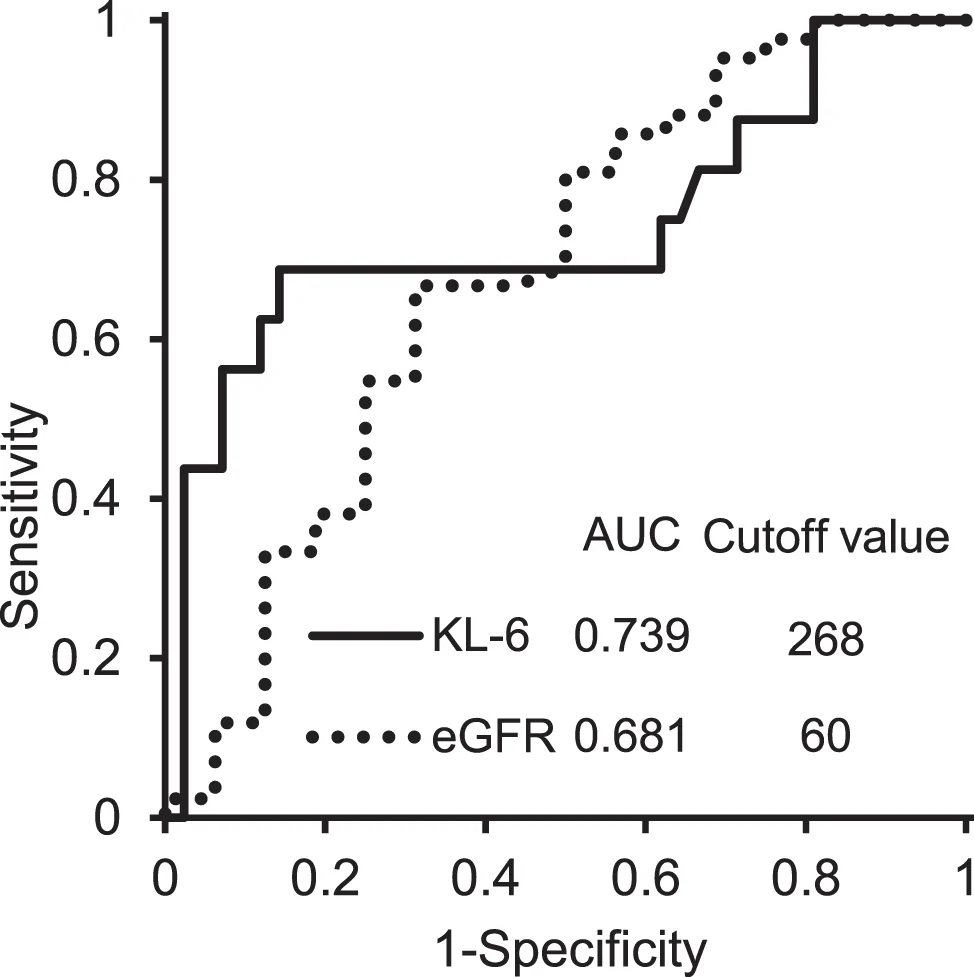
Receiver operating characteristic (ROC) curve for KL-6 elevation. ROC curve analysis was used to determine the optimal cutoff values for baseline serum KL-6 and eGFR levels associated with KL-6 elevation (≥500 U/mL). KL-6: Krebs von den Lungen-6, eGFR: estimated glomerular filtration rate, ROC: receiver operating characteristic

**Fig. 4:**
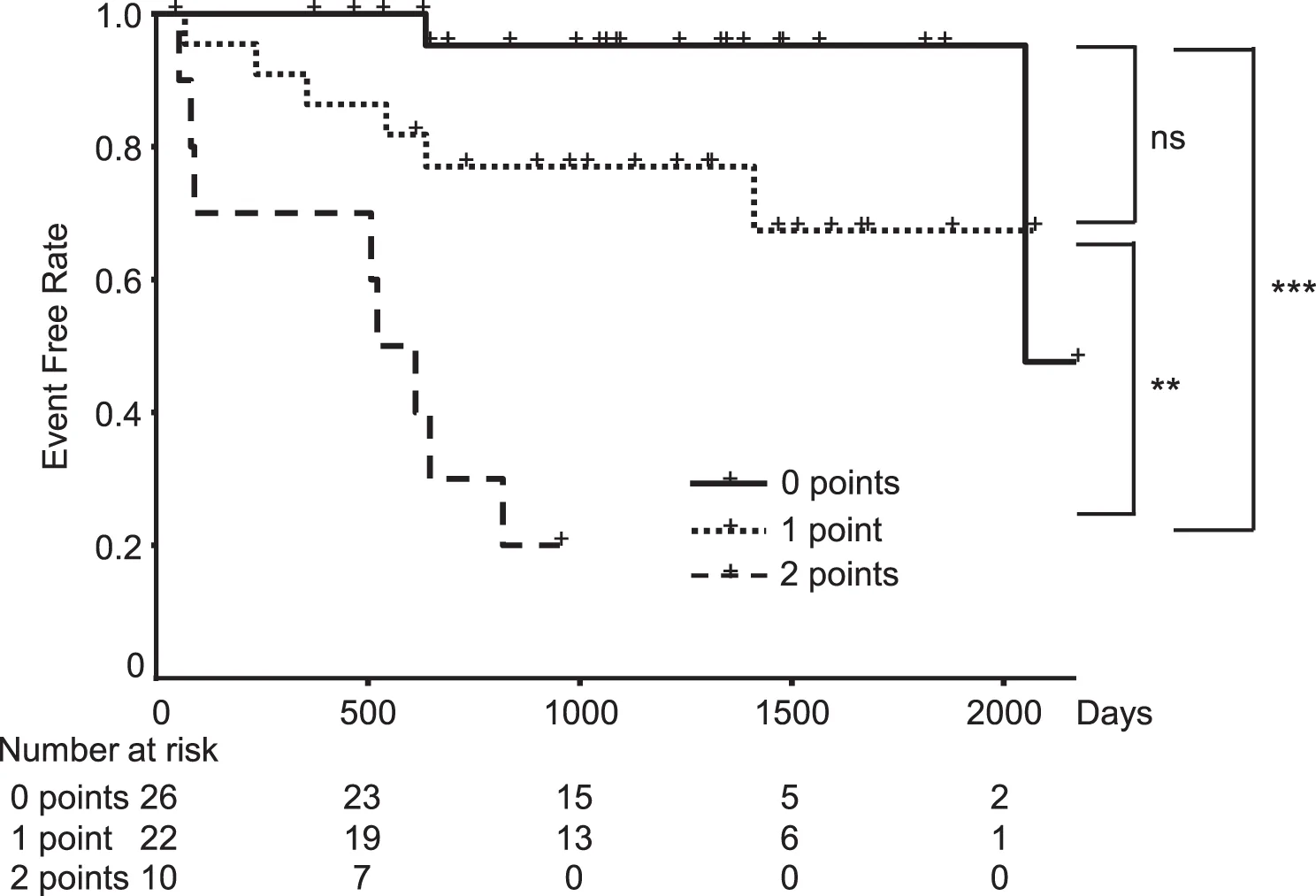
Kaplan–Meier curve of event–free survival based on risk scores. Kaplan–Meier analysis comparing event–free survival among patients stratified by risk score (0, 1, or 2 points). A risk score of 1 was assigned for each of the following factors: baseline KL-6 level > 268 U/mL and eGFR < 60 mL/min/1.73 m^2^. The occurrence of serum KL-6 ≥ 500 U/mL was defined as the event. ns: not significant, **: *p* < 0.01, ***: *p* < 0.001

As shown in Table 4, six factors (baseline KL-6 level, Cre, eGFR, sex, age, and smoking) exhibited *p* < 0.1 in the comparison between the KL-6 elevated and stable groups. Among these factors, Cre was excluded from the multivariable analysis to avoid multicollinearity between Cre and eGFR. Consequently, five factors were included in the multivariable analysis: among them, baseline KL-6 and eGFR were identified as independent factors for KL-6 elevation. The adjusted odds ratios were 1.016 (95% confidence interval (CI): 1.005–1.027, *p* = 0.004) for baseline KL-6 and 0.929 (95% CI: 0.876–0.984, *p* = 0.012) for eGFR. ROC analysis revealed that the cutoff values for baseline KL-6 and eGFR were 268 U/mL and 60 mL/min/1.73 m^2^, respectively (Fig. 3). Based on these cutoff values, risk scores were assigned to each patient (see Additional file 1: Supplementary Table S1). As shown in Table 5, patients in the KL-6 elevated group tended to have higher risk scores. The estimated event (KL-6 elevation)-free survival differed significantly between patients with scores of 0 and 2 (*p* < 0.001) and between patients with scores of 1 and 2 (*p* < 0.01), whereas no significant difference was observed between patients with scores of 0 and 1 (*p* = 0.262) (Fig. 4).

**Table 4:** Multivariable logistic regression analysis of factors associated with KL-6 elevation

Covariates	KL-6 elevated (n = 16)	KL-6 stable (n = 42)	Crude OR (95%CI)	*p* **value**	Adjusted OR (95%CI)	*p* **value**
Baseline KL-6 level [U/mL]	300 (152–371)	167 (127–214)	1.008 (1.002–1.014)	0.007	1.016 (1.005–1.027)	0.004
eGFR [mL/min/1.73 m^2^]	57.4 (43.9–65.0)	64.3 (57.2–83.6)	0.969 (0.933–0.999)	0.039	0.929 (0.876–0.984)	0.012
Female, n	6	5	4.440 (1.120–17.60)	0.034	1.717 (0.152–19.35)	0.662
Age [years]	33 (25–48)	45 (36–51)	0.947 (0.899–0.996)	0.036	0.923 (0.850–1.001)	0.054
Non-smoker, n	13	22	3.939 (0.977–15.88)	0.054	12.75 (0.788–206.1)	0.073

Results of multivariable logistic regression analysis identifying independent risk factors for KL-6 elevation (≥ 500 U/mL). Baseline KL-6 and eGFR were significantly associated with KL-6 elevation, with corresponding odds ratios and confidence intervals shownKL-6: Krebs von den Lungen-6, eGFR: estimated glomerular filtration rate, OR: odds ratio, CI: confidence interval

**Table 5:** Risk score stratification based on baseline KL-6 and eGFR levels

Risk score	KL-6 elevated (n = 16)	KL-6 stable (n = 42)
0 points	2	24
1 point	6	16
2 points	8	2

Patients were assigned a score of 1 point each for baseline KL-6 > 268 U/mL and eGFR < 60 mL/min/1.73 m². Risk scores (0–2 points) were used to stratify patients. KL-6: Krebs von den Lungen-6, eGFR: estimated glomerular filtration rate

### Clinical course after KL-6 elevation

Among 16 patients with KL-6 elevation, EVR was reduced or discontinued in 7 cases. Serum KL-6 levels decreased in 6 of 7 patients, whereas 7 of 9 patients who continued EVR at the same dose exhibited stable or increased KL-6 levels (Table 6).

**Table 6:** Changes in serum KL-6 levels according to EVR dose modification after KL-6 elevation

Action	KL-6 decreased	KL-6 unchanged	KL-6 increased	**not assessed**^**a**^
Discontinued	2	0	0	0
Dose reduced	4	1	0	0
Continued	1	4	3	1

Changes in serum KL-6 levels following continuation, dose reduction, or discontinuation of EVR after KL-6 elevation. Changes in KL-6 levels were categorized as decreased, unchanged, or increased based on subsequent measurements after EVR dose modification.KL-6: Krebs von den Lungen-6, ^a^: not assessed due to insufficient KL-6 measurement

CT imaging tests were performed for 5 of 16 patients classified as KL-6 elevated group. Among them, 2 patients exhibited interstitial changes, including 1 patient with reduced diffusing capacity of the lung for carbon monoxide. EVR was discontinued in both patients, and subsequent improvement in clinical findings and reduction of serum KL-6 levels were observed. The remaining 3 patients showed no radiological abnormalities. Of 3 patients without radiological abnormalities at the time of KL-6 elevation, 1 patient remained asymptomatic during the follow-up period, while the remaining 2 patients required subsequent dose adjustment based on KL-6 trends. Among 11 patients who did not undergo CT examination, none was diagnosed with symptomatic interstitial lung disease during the observed follow-up period. 

## Discussion

This study revealed that EVR administration is associated with KL-6 elevation in heart transplant recipients. Furthermore, elevated serum KL-6 and decreased eGFR before EVR initiation were independent risk factors associated with KL-6 elevation. Because patients with these two risk factors are at a higher risk of KL-6 elevation, careful monitoring is warranted when administering EVR to such patients.

In this study, a significantly higher proportion of patients in the EVR group experienced a KL-6 elevation (27.6% [16/58]) than in the non-EVR group (6.7% [1/15]). Notably, one patient in the non-EVR group with KL-6 elevation was diagnosed with hypersensitivity pneumonitis. These findings suggest that serum KL-6 levels may increase after EVR initiation. KL-6 is widely utilized in the diagnosis of interstitial pneumonia and in evaluating disease progression, boasting a high sensitivity (93.9%) and specificity (96.3%) [19–21]; however, studies regarding its applicability to drug-induced LI are limited. Although several previous studies have reported that serum KL-6 increased in approximately 50% of patients with drug-induced LI, suggesting its potential utility as a reference marker, the diagnostic value of KL-6 for drug-induced LI remains under debate [22, 23]. In addition, drug-induced LI is typically diagnosed based on a comprehensive assessment incorporating clinical symptoms, laboratory findings, and imaging studies [23]. Taken together, serum KL-6 alone is not sufficient for definitive diagnosis but may provide supportive information for detecting drug-induced LI. In our cohort, 5 patients with KL-6 elevation underwent CT imaging, of whom 2 demonstrated interstitial changes. Among these 2 patients, 1 exhibited reduced diffusing capacity of the lung for carbon monoxide. The remaining 3 showed no radiological abnormalities or accompanying symptoms; therefore, they were not diagnosed with LI by the attending physician. In addition, although 37.5% (6/16) of patients in KL-6 elevated group had a history of cytomegalovirus antigenemia, no patients were clinically diagnosed with cytomegalovirus pneumonitis at the time of peak KL-6. These findings suggest that KL-6 may reflect subtle pulmonary changes not identified by imaging or symptoms. Taken together, KL-6 may serve as a reference biomarker that contributes to the clinical assessment of potential drug-induced LI in patients treated with EVR, although it should be interpreted together with clinical symptoms and imaging findings.

We identified elevated serum KL-6 and decreased eGFR before EVR initiation as independent risk factors for EVR-associated KL-6 elevation. This observation is consistent with that reported for patients with cancer [17]. There are several possible causes for elevated serum KL-6 levels. First, serum KL-6 levels are known to increase with cancer progression; however, the mean baseline serum KL-6 level in the KL-6 elevated group (282 U/mL) was lower than the mean serum KL-6 level reported in patients with cancer who developed drug-induced LI (438 U/mL). Notably, none of the patients in our study had a history of LI or cancer requiring treatment at the time of transplantation or EVR initiation. Second, prior to transplantation, heart transplant recipients often take amiodarone, a drug with a high potential to cause LI [24]. However, the proportion of patients with a history of amiodarone use was approximately 50% in both the KL-6 elevated group and KL-6 stable group. Although the cause of high serum KL-6 levels before EVR initiation remains unclear, elevated serum KL-6 levels may be a common risk factor for both transplantation and cancer. This suggests that high serum KL-6 levels indicate the presence of minor underlying lung vulnerability.

In this study, a decreased eGFR was identified as an independent risk factor for KL-6 elevation. This observation appears to have important implications for the selection of immunosuppressive agents because EVR is often introduced to prevent the deterioration of renal function. However, renal function should be interpreted with caution in patients with heart failure because patients with advanced heart failure may develop sarcopenia, potentially leading to overestimation of eGFR based on serum creatinine levels. In particular, muscular dystrophy, a potential underlying cause of heart failure [25], may further contribute to this bias through reduced muscle mass and deceptively low Cre levels. In our cohort, three patients with muscular dystrophy were included in the KL-6 stable group; however decreased eGFR was an independent risk factor and it remained even after excluding these patients from the analysis (cutoff value: 60 mL/min/1.73 m^2^, *p* = 0.031). This finding suggests that impaired renal function may contribute to KL-6 elevation. Consistent with our observation, Weiner et al. reported that reduced renal function was a risk factor for LI associated with sirolimus, an mTOR inhibitor, similar to EVR [26]. However, the underlying mechanism by which impaired renal function increases the risk of mTOR inhibitor–associated KL-6 elevation remains unclear and is likely multifactorial. One possible explanation is fluid retention associated with renal dysfunction, which may contribute to subclinical pulmonary congestion and subsequent KL-6 elevation. In the present study, all patients in the KL-6 elevated group underwent chest CT or chest X-ray examinations, and no apparent signs of pulmonary congestion or significant pleural effusion were observed at the time of KL-6 elevation. In addition, hemodynamic parameters obtained from RHC were relatively low and not suggestive of overt volume overload (Table 3); however, these parameters were not necessarily measured at the same time as KL-6 elevation and varied among patients. Therefore, the contribution of subclinical pulmonary congestion cannot be fully excluded. Another possible explanation is increased drug exposure. Previous studies have reported dose-dependent cellular toxicity of mTOR inhibitors, although clinical evidence regarding the association between EVR concentration and LI risk remains inconsistent [12, 13]. In our cohort, the mean EVR concentration tended to be higher in the KL-6 elevated group than KL-6 stable groups (6.5 [5.7–7.6] vs 5.6 [3.9–6.6] ng/mL, *p* = 0.115). Furthermore, serum KL-6 levels decreased in 6 of 7 patients after dose reduction or discontinuation of EVR. In contrast, 7 of 9 patients who continued EVR at the same dose exhibited stable or increased serum KL-6 levels (Table 6). In addition, EVR discontinuation was followed by improvement in clinical findings and reduction of serum KL-6 levels in the 2 patients exhibited interstitial changes on CT examination. These findings may suggest a possible relationship between EVR exposure and KL-6 elevation. However, because EVR is primarily metabolized in the liver, it is unlikely that EVR exposure is directly affected by renal function itself. Therefore, the mechanism underlying the association between renal dysfunction and KL-6 elevation remains unclear, and further studies are warranted.

Although not statistically significant, a trend toward an increased risk of KL-6 elevation was observed in younger patients and nonsmokers. Typically, older age and smoking history are recognized as risk factors for LI [24, 27]. Therefore, the contrary trend identified in our study needs to be interpreted with caution. One possible explanation is the age restriction, transplant candidates must be 65 years old or younger at the time of registration, resulting in a study cohort predominantly composed of individuals in their forties. As immune function declines with age, younger patients may be more susceptible to immune-mediated KL-6 elevation; however, this remains speculative. With regard to smoking history, all patients had ceased smoking while awaiting transplantation, and none had smoked within the five years preceding transplantation. Thus, the influence of remote smoking history on the KL-6 elevation in this cohort was minimal.

We calculated a risk score based on the cutoff values of serum KL-6 and eGFR and found that higher risk scores were associated with a higher incidence of KL-6 elevation. In addition, patients with a score of 2 experienced significantly earlier KL-6 elevation than those with scores of 0 or 1 (Fig. 4). The time of onset of EVR-associated LI in cancer patients is reportedly 2–3 months after administration [17]. In contrast, among patients who developed EVR-associated LI after renal transplantation, 62.5% developed EVR-associated LI > 6 months post-transplantation, with a median onset time of 752 days [range, 32–1502 days] [12]. Similarly, in this study, 10 of 16 patients with EVR-associated KL-6 elevation developed more than 12 months after EVR initiation, with a median onset time of 533 days [range: 54–2051 days]. This difference in the onset time between cancer and transplant patients could be explained by the difference in dosage. In renal cell carcinoma and breast cancer, EVR is typically administered at 10 mg, nearly ten times higher than the dose used in transplant recipients [28]. This substantial difference in dosage may have contributed to the earlier onset of EVR-associated KL-6 elevation in patients with cancer. Another possible explanation is the difference in co-medication, specifically immunosuppressive drugs such as steroids. Transplant recipients routinely use corticosteroids (e.g., prednisolone) concomitantly with EVR, which may mitigate the early onset of EVR-associated KL-6 elevation owing to their strong immunosuppressive effects. When initiating EVR, long-term continuous patient monitoring is necessary to manage EVR-associated KL-6 elevation, particularly in transplant recipients.

Table 6 shows the changes in KL-6 levels following EVR dose modification. 6 of 7 patients who underwent dose reduction or discontinuation showed a decrease in KL-6 levels, whereas 7 of 9 patients who continued EVR tended to show persistent elevation or further increases in KL-6 levels. These findings suggest that KL-6 levels changes in exposure–response manner and that KL-6 elevation may serve as an early indicator prompting closer monitoring or timely modification of EVR therapy.

This study had some limitations. First, this was a single-center retrospective study with a limited sample size and may have contained potentially undetectable factors. However, this study is the first to identify the risk factors for KL-6 elevation in heart transplant recipients, offering valuable insights for future research and clinical practice. Second, EVR-associated LI was evaluated primarily based solely on serum KL-6 levels. Although KL-6 is known as a sensitive biomarker for interstitial lung diseases [19–21], its diagnostic value for drug-induced LI remains controversial; therefore, the possibility of over- or under-diagnosis in this study cannot be excluded. In addition, although no patients were diagnosed with cytomegalovirus pneumonitis, subclinical infection could not be excluded. Third, owing to the retrospective design, the timing and frequency of serum KL-6 measurements after EVR administration and the duration of follow-up were inconsistent across our study cohort. In addition, the decision to initiate EVR may have been influenced by underlying clinical characteristics, resulting in potential confounding by indication. Finally, because renal function was estimated using Cre-based formula, renal function may have been overestimated in some patients with reduced muscle mass or sarcopenia. Therefore, the cutoff value of eGFR calculated in this study were optimized for our specific dataset and may not be directly generalizable to other populations. Future external validation in independent cohorts is required.

## Conclusion

In this study, we demonstrated that EVR administration was associated with an increased risk of KL-6 elevation in heart transplant recipients. Furthermore, elevated baseline serum KL-6 levels and impaired renal function were identified as independent predictors of KL-6 elevation. Patients with both factors may be at higher risk. Therefore, when initiating EVR treatment in heart transplant recipients, particularly in those with elevated serum KL-6 levels and renal dysfunction, serum KL-6 levels should be closely monitored, and imaging tests may be considered in response to changes in KL-6 levels.

## Electronic supplementary material

Below is the link to the electronic supplementary material.

Supplementary Material 1

## Data Availability

The datasets generated and/or analyzed during the current study are not publicly available due to privacy concerns and institutional policy but are available from the corresponding author on reasonable request.

## Abbreviations

EVR: Everolimus
mTOR: Mammalian target of rapamycin
SOT: Solid organ transplantation
LI: Lung injury
KL-6: Krebs von den lungen-6
eGFR: Estimated glomerular filtration rate
Cre: Creatinine
ROC: Receiver operating characteristic
CI: Confidence interval
