# Supplementary material for: Serum KL-6 and eGFR predict everolimus-associated KL-6 elevation in heart transplant recipients: a retrospective observational study
Source: J Pharm Health Care Sci. 2026 Jul 1;12:83. doi: 10.1186/s40780-026-00603-0 (PMC13599193; doi:10.1186/s40780-026-00603-0)
Supplement: Supplementary file 1 [file 40780_2026_603_MOESM1_ESM.docx]

Supplementary Table S1. Patient characteristics at the time of EVR initiation stratified by risk score

|  | 0 points (n = 26) | 1 point (n = 22) | 2points (n = 10) |
| --- | --- | --- | --- |
| Age [years], median (IQR) | 43 (33 – 48) | 45 (33 – 53) | 49 (41 – 51) |
| Male, n (%) | 24 (92.3) | 17 (77.3) | 6 (60.0) |
| BMI [kg/m^2^], median (IQR) | 20.3 (18.6 – 21.8) | 19.1 (17.5 – 22.8) | 22.4 (20.0 – 23.6) |
| History of smoking, n (%) | 9 (34.6) | 9 (40.9) | 5 (50.0) |
| Calcineurin Inhibitors, n (%) | | | |
| Tacrolimus | 15 (57.7) | 11 (50.0) | 6 (60.0) |
| Ciclosporin | 11 (42.3) | 11 (50.0) | 4 (40.0) |
| History of steroid pulse therapy, n (%) | 16 (61.5) | 12 (54.5) | 3 (30.0) |
| History of amiodarone, n (%) | 14 (53.8) | 10 (45.5) | 5 (50.0) |
| Time to EVR initiation [days], median (IQR) | 96 (81 – 265) | 134 (85 – 358) | 130 (90 – 174) |
| Laboratory data, median (IQR) | | | |
| AST [U/L] | 17 (15 – 26) | 24 (14 – 27) | 22 (17 – 28) |
| ALT [U/L] | 15 (12 – 27) | 18 (11 – 26) | 19 (12 – 37) |
| T-Bil [mg/dL] | 0.6 (0.5 – 0.7) | 0.5 (0.4 – 0.9) | 0.5 (0.4 – 0.7) |
| LDH [U/L] | 271 (199 – 339) | 260 (201 – 320) | 263 (221 – 303) |
| Albumin [g/dL] | 4.3 (3.8 – 4.5) | 4.2 (3.7 – 4.4) | 4.2 (3.8 – 4.3) |
| CRP [mg/dL] | 0.04 (0.02 – 0.10) | 0.05 (0.02 – 0.18) | 0.04 (0.02 – 0.09) |
| BUN [mg/dL] | 17.6 (12.9 – 21.5) | 19.0 (16.6 – 21.6) | 22.6 (16.3 – 27.0) |
| Creatinine [mg/dL] | 0.90 (0.71 – 0.97) | 1.11 (0.90 – 1.32) | 1.26 (1.16 – 1.44) |
| eGFR [mL/min/1.73m^2^] | 78.1 (64.7 – 97.4) | 57.3 (54.4 – 63.9) | 46.7 (41.3 – 53.1) |
| White blood cell [/µL] | 5400 (3700 – 6800) | 4450 (3700 – 6400) | 3850 (2300 – 5300) |
| Neutrophil [/µL] | 4057 (2862 – 5501) | 3729 (2260 – 4762) | 2746 (1615 – 4357) |
| Eosinophil [/µL] | 45 (26 – 80) | 33 (12 – 72) | 35 (19 – 50) |
| Hemoglobin [g/dL] | 12.4 (11.6 – 13.0) | 11.6 (10.8 – 12.8) | 11.9 (10.2 – 12.7) |
| Platelet [×10^4^/µL] | 23.2 (19.1 – 26.9) | 21.2 (18.5 – 25.9) | 21.0 (19.9 – 27.5) |
| BNP [pg/mL] | 89.5 (48.0 – 129.8) | 104.1 (46.4 – 156.8) | 96.3 (46.7 – 152.0) |
| Baseline KL–6 level [U/mL],  median (IQR) ^a^ | 157 (119 – 187) | 164 (131 – 282) | 368 (289 – 418) |

EVR: everolimus, AST: aspartate aminotransferase, ALT: alanine aminotransferase, T-bil; total bilirubin, LDH: lactate dehydrogenase, CRP: C-reactive protein, BUN: blood urea nitrogen, eGFR: estimated glomerular filtration rate, BNP: B‐type natriuretic peptide, KL-6: Krebs von den Lungen-6

^a^: Baseline KL-6 levels were defined as those measured before the EVR initiation
